# Mandibular Mobility as a Marker for Temporomandibular Joint Arthritis in Juvenile Idiopathic Arthritis—A Cross-Sectional Observational Study

**DOI:** 10.3390/jcm14207385

**Published:** 2025-10-19

**Authors:** Tamara Pawlaczyk-Kamieńska

**Affiliations:** Department of Pediatric Dentistry, Poznan University of Medical Sciences, Ul. Bukowska 70, 60-812 Poznań, Poland; tpawlaczyk@ump.edu.pl

**Keywords:** temporomandibular joint, juvenile idiopathic arthritis, Cone-Beam Computed Tomography, Cross-Sectional Studies

## Abstract

**Background/Objectives:** This study aims to evaluate the functional characteristics of the temporomandibular joint (TMJ) in patients diagnosed with juvenile idiopathic arthritis (JIA). Specifically, it seeks to determine the prevalence of TMJ involvement and its impact on clinical symptoms and functional limitations. **Methods:** A total of 40 patients diagnosed with JIA according to the International League of Associations for Rheumatology criteria were included. Exclusion criteria encompassed systemic diseases unrelated to JIA, prior craniofacial surgery, and trauma. Participants were divided into two groups: those with TMJ arthritis (n = 23) and those without (n = 17). Clinical assessments were conducted using the Helkimo anamnestic index (Ai) and dysfunction index (Di) to evaluate TMJ-related symptoms. **Results:** The Ai revealed that most patients reported no or only mild subjective symptoms. Overall Di distributions did not differ significantly between groups, although mandibular mobility was more impaired in the TMJ arthritis group. A moderate correlation (r = 0.4) was observed between Ai and Di. **Conclusions:** This pilot study indicates that impaired mandibular mobility may suggest TMJ involvement in JIA. Given the study’s limitations, such as being a single-center study with a small sample size and using a CBCT-based classification, further multicenter studies utilizing validated pediatric protocols are necessary to reinforce these preliminary findings.

## 1. Introduction

Juvenile idiopathic arthritis (JIA) is the most common arthropathy affecting children and adolescents [1,2] and can occur at any stage of development. The incidence of JIA among children is 0.03%, and it occurs more often in girls (0.02%) than in boys (0.01%) [3]. The disease has an onset before 16 years of age and a duration of at least 6 weeks, and its diagnosis is reached after excluding other known causes of arthritis [2,3,4]. The disease is a chronic inflammatory process of unknown etiology, frequently of autoimmune origin, that damages the joint cartilage and bone epiphysis. During the disease course, inflammatory changes affect joints with a synovial membrane. The polyarticular form refers to JIA that affects more than four joints, while the oligoarticular form refers to JIA that affects up to four joints [1,3,4]. Temporomandibular joint (TMJ) involvement often accompanies the involvement of other synovial joints. However, the TMJ can sometimes be the only disease site, with one or both joints affected [5]. In 17–50% of JIA patients diagnosed with TMJ arthritis, changes are observed in only one joint, although it is believed that the disease may begin in one joint and eventually progress to the other [5,6,7,8,9].

In many cases, TMJ arthritis occurs without clinical or subjective symptoms, which can delay the detection of inflammation in the affected joint [5,7,8,9,10]. However, TMJ arthritis can have serious consequences. Specifically, it can lead not only to joint dysfunction, but also to disruption or inhibition of mandibular growth and development by causing damage to the mandibular growth center located in the condylar process [11,12]. Therefore, TMJ arthritis can have irreversible functional and/or esthetic effects in the craniofacial region, such as facial asymmetry, micrognathia, retrognathia, malocclusion, and ankylosis of the TMJ [12]. The potential for irreversible effects underscores the need for urgent action in the diagnosis and treatment.

It is essential to achieve early diagnosis and treatment of JIA. The incidence of TMJ arthritis in JIA patients has been reported to range from 17 to 87% [5,6,7,12]. This wide range arises from the influence of many factors, including different study population sizes, different research methods and non-standard diagnostic criteria used by researchers, and difficulties in conducting research because of the young age of the patients and their level of cooperation, as well as the disease duration, type, and activity (periods of remission or exacerbation) [12,13,14,15,16]. In this study, we defined TMJ arthritis using findings from cone-beam computed tomography (CBCT) of the condylar processes. While we recognize that MRI is considered the gold standard for detecting early inflammatory changes, we chose CBCT due to its greater availability and effectiveness in identifying structural alterations.

## 2. The Aim of the Study

The aim of this study was to systematically analyze the functional characteristics of the TMJ in patients diagnosed with JIA.

## 3. Material and Methods

The study involved 40 children who had a confirmed diagnosis of Juvenile Idiopathic Arthritis, according to the criteria set by the International League of Associations for Rheumatology [2]. At the time of assessment, all participants were in a clinically stable phase of their disease. At the time of inclusion, all patients were receiving rheumatologic care and appropriate systemic therapy. None of the participants were completely ‘healed’ in terms of achieving remission without treatment; however, all were in a controlled or stable phase of the disease and not experiencing any acute inflammatory flare-ups. The exclusion criteria included: 1. acute systemic exacerbations or active extra-articular manifestations; 2. any systemic disease other than JIA; 3. prior surgical procedures in the craniofacial region; and 4. previous craniofacial trauma. Based on the findings of cone beam computed tomography (CBCT) examinations of the TMJ condylar processes, the included JIA patients were divided into two groups: TMJ arthritis patients (n = 23; 57.5%) and non-TMJ arthritis patients (n = 17; 42.5%).

CBCT examinations were conducted using the Scanora 3D XL (Soredex, Tuusula, Finland) while the subjects were in an upright position with their jaws closed. The radiographic data were then reconstructed into corrected sagittal and axial images of the condyles using OnDemand 3D (CyberMed, Seoul, Republic of Korea) for further analysis. A modified radiological grading system developed by Billiau et al. (2007) [17] was employed to assess the morphology of the condylar process. This system categorizes the appearance as follows: a radiologically normal appearance is scored as 0, cortical bony erosions as 1, flattening as 2, condylar flattening with additional erosions as 3, and a complete loss of the condyle as 4 [17]. All CBCT scans were independently reviewed by an experienced oral radiologist. It is important to emphasize that CBCT primarily detects structural sequelae rather than active inflammatory changes.

All patients underwent a clinical examination. The Helkimo anamnestic index (Ai) and Helkimo dysfunction index (Di) [18] were applied to assess subjective and objective symptoms of TMJ dysfunction. Although the Helkimo index is a historically widely used diagnostic tool—especially in epidemiological studies—it has known limitations such as low specificity, limited differentiation of symptom sources (e.g., muscular vs. articular), and lack of validation in pediatric populations. Therefore, in interpreting our findings, we also referred to the contemporary standardized Clinical Orofacial Examination Protocol by Stoustrup et al. [15], which is increasingly recommended in pediatric rheumatology and dentistry for assessing TMJ involvement in JIA patients.

For the Ai value, self-reported anamnestic data regarding TMJ symptoms were collected using a standardized questionnaire addressed to the patients and/or their parents or guardians. Based on the answers obtained, an appropriate Ai value was assigned according to an anamnestic scale [18], as follows: Ai 0, no subjective symptoms of TMJ dysfunction; Ai I, mild symptoms including sensation of jaw fatigue, jaw stiffness, and TMJ sounds (clicking or crepitus); and Ai II, severe symptoms including one or more of the following: difficulty in mouth opening, jaw locking, dislocation and painful movement of the mandible, pain in the TMJ region and/or masticatory muscles.

The Di index evaluates five items (mandibular mobility, TMJ function, muscle pain, TMJ pain, and pain on movement of the mandible) using a three-point scale of increasing severity: 0, no symptoms; 1, moderate symptoms; and 5, advanced symptoms. In this study, the maximum mouth opening (MMO) scale proposed by Müller et al. [19] was used. For this, reduced range of MMO was defined as <35 mm in children aged <10 years and <40 mm in children aged ≥10 years. For lateral movements, we proposed preliminary cut-off values (<7 mm in patients ≥10 years; <6 mm in patients <10 years). These thresholds were exploratory, not validated, and should be interpreted with caution. TMJ function was scored based on presence of joint sounds or locking (0/1/5). Muscle pain and TMJ tenderness were scored on palpation (0/1/5). Pain on movement was evaluated during maximum opening and lateral movements (0/1/5). Based on the clinical results, the Di value [18] was calculated. The sum (range: 0–25 points) of the individual scores constitutes the dysfunction score and forms the basis of the clinical dysfunction index. Symptoms detected during the clinical examination were expressed as follows: Di 0 (0 points; no symptoms); Di I (1–4 points; mild symptoms); Di II (5–9 points; moderate symptoms); and Di III (10–25 points; severe symptoms). The Helkimo index (1974) is a historically widely used diagnostic tool, particularly in epidemiological studies. Therefore, in interpreting our findings, we also referred to the standardized Clinical Orofacial Examination Protocol by Stoustrup et al. [15], which are increasingly recommended in pediatric rheumatology and dentistry for assessing TMJ involvement in JIA patients

Patients were consecutively recruited from the Rheumatology Clinic. This study was designed as a pilot exploratory investigation; no formal sample size calculation was conducted. All participants aged > 16 years and their legal guardians received an explanation of the purpose and process of the study and subsequently provided written informed consent to participate. With the consent of the patients’ legal guardians, data on the general disease characteristics were obtained from records in the Rheumatology Clinic. The research was conducted in accordance with ethical principles, including the World Medical Association Declaration of Helsinki. The experimental protocol was approved by the Ethical Committee of the Poznan University of Medical Sciences, Poland (No. 1255/18).

This study was conducted as a pilot exploratory investigation, and therefore, no formal sample size calculation was performed. The final sample size (n = 40) consisted of all eligible patients who were treated during the recruitment period. The relatively small sample size may reduce the statistical power and generalizability of the results, which is acknowledged as a limitation of this study.

## 4. Statistical Analysis

Statistical analyses were conducted using Statistica software (version 12, StatSoft, Inc., Tulsa, OK, USA). Descriptive statistics were calculated for all variables. To compare quantitative data between the TMJ arthritis group and the non-TMJ arthritis group, the Mann–Whitney U test was utilized. Effect sizes (calculated as r = Z/√N) were determined for non-parametric comparisons, and 95% confidence intervals (CIs) were reported to assess the precision of the estimates. Qualitative variables were compared using either the chi-squared test or Fisher’s exact test. The relationship between the subjective index (Ai) and the objective index (Di) was evaluated using Spearman’s rank correlation coefficient, along with a 95% CI. Additionally, receiver operating characteristic (ROC) curve analysis was performed to assess the discriminative capacity of mandibular mobility in identifying TMJ arthritis and to determine optimal cutoff values. A *p*-value of less than 0.05 was considered statistically significant.

## 5. Results

Girls constituted 67.5% of the JIA patients and showed similar percentages among the TMJ arthritis patients (70%) and non-TMJ arthritis patients (65%). The mean age of the JIA patients was 12.88 ± 3.39 years, the mean disease duration was 3.95 ± 2.40 years, and the mean age at diagnosis was 8.93 ± 3.68 years. The oligoarticular form was noted in 47.5% of the patients. The above parameters showed no significant differences (*p* ≥ 0.05) between the TMJ arthritis and non-TMJ arthritis patients (Table 1).

**Figure 1:**
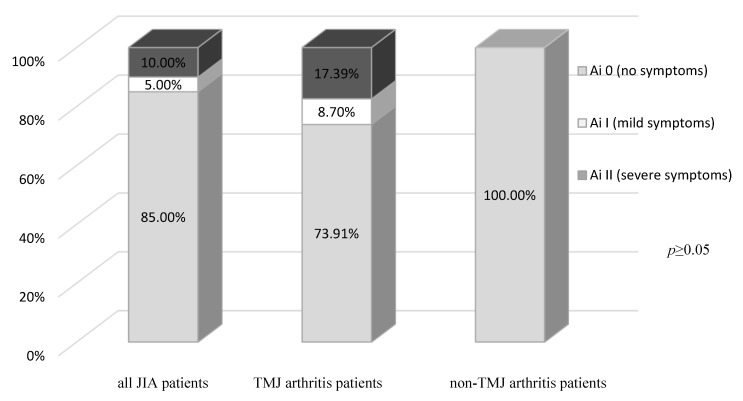
Anamnestic index (Ai) in all JIA patients, and TMJ arthritis and non-TMJ arthritis patients.

The percentage of JIA patients without subjective TMJ dysfunction symptoms (Ai 0) was 85%. 5% reported mild (Ai I) and 10% severe subjective TMJ symptoms (Ai II). The Ai values did not differ significantly (*p* ≥ 0.05) between the TMJ arthritis and non-TMJ arthritis patients (Figure 1).

**Figure 2:**
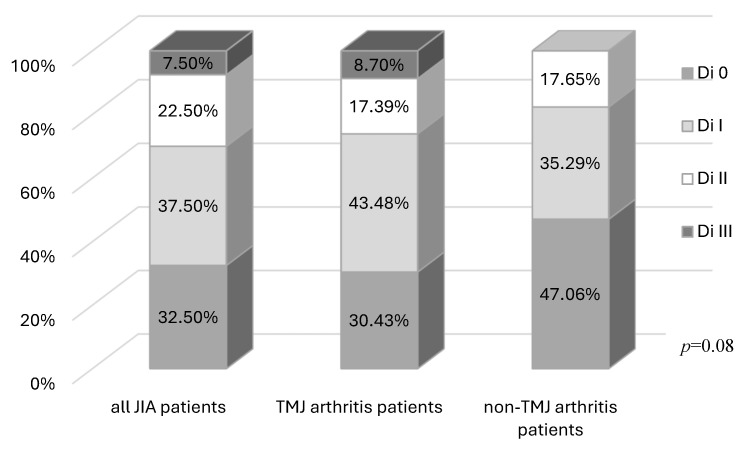
Clinical index (Di) in all JIA patients, and TMJ arthritis and non-TMJ arthritis patients.

Absence of clinical TMJ dysfunction symptoms (Di 0) was noted in 32.5% of the JIA patients. Mild symptoms (Di I) were observed in 37.5%, moderate (Di II) in 22.5%, and severe (Di III) in 7.5%. There was no significant difference (*p* = 0.08) between the TMJ arthritis and non-TMJ arthritis patients (Figure 2).

**Figure 3:**
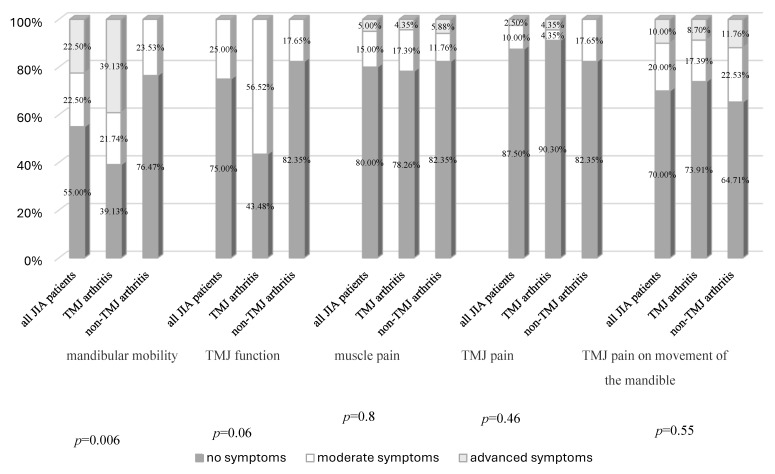
Components of the Clinical index (Di) in all JIA patients, and TMJ arthritis and non-TMJ arthritis patients.

Then the individual components of the clinical index (Di) were evaluated, a significant difference (*p* = 0.005) was observed between the percentages of TMJ arthritis and non-TMJ arthritis patients for the mandibular mobility values. A normal amplitude of mandibular mobility was noted in 55% of the JIA patients (39.13% of TMJ arthritis patients versus 76.47% of non-TMJ arthritis patients). Moderate disturbance was observed in 22.5% of the JIA patients (21.74% of TMJ arthritis patients versus 23.53% of non-TMJ arthritis patients). Severe disturbance was found in 22.5% of the JIA patients, and this level of dysfunction in mandibular mobility was noted only in TMJ arthritis patients (39.13%) (Figure 3). The other four components of the Di, namely TMJ function, muscle pain, TMJ pain, and pain on movement of the mandible, did not show any significant differences (*p* ≥ 0.05) between the percentages of affected TMJ arthritis and non-TMJ arthritis patients (Figure 3).

A moderate positive correlation was observed between the Ai index and the Di index in JIA patients (r = 0.4, 95% CI [0.17–0.74], *p* = 0.003). This indicates that higher self-reported symptom severity is associated with greater objective dysfunction. A similar moderate positive correlation was found between the Ai value and the amplitude of mandibular mobility (r = 0.4). Additionally, the difference in mandibular mobility between TMJ arthritis and non-TMJ arthritis patients was statistically significant (U = 286.5, *p* = 0.006, r = 0.39, 95% CI [0.10–0.69]), suggesting a moderate effect size. This finding supports the hypothesis that reduced mandibular mobility may serve as a functional indicator of TMJ involvement in children with JIA. However, receiver operating characteristic (ROC) curve analysis demonstrated limited discriminatory ability of mandibular mobility as an isolated predictor of TMJ arthritis (AUC = 0.27, 95% CI [0.12–0.43]), and no clinically meaningful cutoff value was identified. These results imply that while functional impairment reflects TMJ pathology, it should not be used alone as a diagnostic discriminator.

To enhance the interpretation of clinical symptoms, the findings were examined in relation to the Clinical Orofacial Examination Protocol proposed by Stoustrup et al. [15], which is increasingly being adopted for pediatric patients with JIA. The observed reduction in mandibular mobility amplitude and the asymmetries noted in patients with confirmed TMJ arthritis align with the functional markers described in this protocol. These markers include restricted mouth opening (less than 35 mm), deviation during opening, and reduced lateral movements. Such objective indicators support the results obtained using the Helkimo indices and provide a contemporary, standardized framework for evaluating orofacial dysfunction in pediatric populations with JIA.

## 6. Discussion

The orofacial consequences of TMJ involvement are diverse, ranging from clinically asymptomatic and only visualized on MRI or X-ray images to dysfunctions and/or morphological deformations that adversely affect the quality of life of patients [15]. It should be emphasized that TMJ arthritis during the course of JIA is often asymptomatic in the initial stages; patients do not complain of any symptoms, and any slight deviations from the normal state may be unnoticeable in clinical examinations [1,5,11,19,20,21].

In the literature, various diagnostic methods have been used to assess symptoms indicating TMJ involvement in JIA patients [4,8,13,22]. The Helkimo indexes (Ai and Di), while once widely used in studies and epidemiological surveys, are now regarded as having limited clinical utility. Their subjectivity and lack of specificity—especially when distinguishing between muscular and joint dysfunction—along with the absence of validation in pediatric populations, diminish their diagnostic effectiveness today. As a result, newer, standardized assessment methods are favored. One such tool is the Clinical Orofacial Examination Protocol proposed by Stoustrup et al. [15]. This protocol is supported by international expert groups and is specifically designed for children with juvenile idiopathic arthritis (JIA). By incorporating elements of the Stoustrup protocol, the clinical assessment of the temporomandibular joint (TMJ) in JIA has improved in terms of detail and accuracy. Its structured format focuses on objective functional markers, such as the range of mandibular opening, deviations, and joint sounds. While it may require more time to implement, this protocol enhances reliability in pediatric diagnostics and can facilitate earlier intervention.

In our study, Helkimo indexes were retained to allow comparison with historical datasets and previously published findings, but interpretation of results was made cautiously, with reference to the more recent clinical standards. Helkimo index has been used in epidemiological studies on TMJ disorders, as well as in studies involving children and adolescents, including JIA patients [10,14,23,24]. In the present study, the Helkimo indexes were used to analyze the subjective and objective symptoms of TMJ dysfunction in JIA patients. Given the limitations of the Helkimo indices, particularly their lack of pediatric validation, our findings should be considered exploratory. Interpretation was supported by referencing the Stoustrup protocol, which offers a more standardized clinical framework.

Despite using the same research methodology, it remains difficult to compare our results with similar results in previous studies because the studies differ in many other respects. For example, there are large fluctuations in the numbers of subjects (26–266), mean ages of the children at the time of the study (7–14 years), disease durations (3–6 years) [10,14,25,26]. In all of the cited studies, girls constituted about 70% of the patients. The sex ratio in the present study was similar, and this ratio is typical for JIA patients.

Available studies using the Helkimo indexes include patients diagnosed with JIA regardless of TMJ involvement [14,25,26] or only JIA patients with TMJ arthritis [10]. Among the JIA patients in the studies that did not determine TMJ involvement, the percentage of subjects who had no subjective symptoms of TMJ dysfunction (Ai 0) ranged from 30.7% to 56% [4,25,26]. In our study, the corresponding percentage was higher at 85%. Furthermore, in the cited studies, mild symptoms (Ai I) were reported in approximately 9%, and severe symptoms (Ai II) in 35–59.4% [14,26]. In our study, these percentages were lower. In the Polizzi et al. [10] study involving only TMJ arthritis JIA patients, absence of subjective symptoms (Ai 0) was noted in 8% of patients, while mild symptoms (Ai I) were noted in 52%, and severe (Ai II) in 40%. In our study, the percentage of patients with TMJ arthritis without subjective symptoms (Ai 0) was higher. However, the percentages of TMJ arthritis patients who complained of mild symptoms (Ai I) or severe symptoms (Ai II) were lower.

In the studies, including patients diagnosed with JIA regardless of TMJ involvement [14,24,25], the percentage of JIA patients without symptoms of TMJ dysfunction (Di 0) ranged from 24% to 35%. Mild symptoms (Di I) were noted in 19–46% of the patients, moderate symptoms (Di II) in 14–34%, and severe symptoms (Di III) in approximately 15 [14,24,25]. In our study, the percentages of JIA patients with no TMJ dysfunction (Di 0), moderate (Di I), and severe (Di II) symptoms were within these ranges. However, the percentage of subjects with severe symptoms (Di III) was lower. In the study by Polizzi et al. [10], an absence of TMJ dysfunction symptoms (Di 0) was noted in 4% of patients, while there were mild symptoms (Di I) in 28%, moderate symptoms (Di II) in 40.0%, and severe symptoms (Di III) in 28.0%. Among the TMJ arthritis patients in our study, the percentages of patients with low Di values (Di 0 and Di I) were higher, while the percentages of patients with high Di values (Di II and Di III) were lower compared with the previous study [10].

Taken together, the findings of our study and previous studies show significant discrepancies in the subjective and objective clinical symptoms of TMJ dysfunction in JIA patients. Many factors influence these differences in the findings. Undoubtedly, the child’s age at disease onset has a significant influence. As the age of the child at JIA onset decreases, the prognosis for the TMJ becomes worse if it is affected [5,6,15]. A higher degree of subjective and clinical dysfunction is also noted in the polyarticular form compared with the oligoarticular form [5,25]. In our study, the statistical analyses did not reveal such correlations. Our study did not account for potential confounding factors such as JIA subtype, age at onset, disease duration, or systemic therapy. Future studies should use multivariate models to better address these influences.

The process of TMJ degeneration during the course of JIA usually proceeds for a long period without any subjective or clinical symptoms of dysfunction. The consequences of this disease process that affects the joints and possibly damages the condylar process, as well as the mandibular growth center located within it, are usually observed after several years of TMJ involvement [11]. There may be various TMJ dysfunctions and possibly unilateral or bilateral underdevelopment of the mandible, leading to dentofacial deformities [16]. In the initial stage of the disease, slight deviations from the normal state may be unnoticeable [9,13,14]. Based on the results of the present study, clinical predictors that may indicate TMJ involvement in JIA patients are disturbed amplitudes of mandibular movements, namely reduced range of maximum opening and reduced range of lateral movements, consistent with findings in other studies [5,13,22]. We noted the correct amplitude of mandibular movements in a significantly lower percentage of TMJ arthritis patients compared with non-TMJ arthritis patients. Different results were obtained by Stoll et al. [6], who found reduced mouth opening in 33% of JIA patients with TMJ arthritis and 21% of patients without TMJ arthritis. However, the difference was not statistically significant. The remaining components of the Helkimo Di [18], namely joint function, muscle pain, and joint pain, were not identified as indicators of TMJ involvement in JIA patients.

The present study demonstrated that JIA children are at risk for TMJ dysfunction. The identified limitations in mobility suggest that clinical practice should include regular monitoring of mandibular function and development. Imaging should be considered when progressive limitations are observed. Based on our findings, we recommend a straightforward clinical decision algorithm that combines functional assessment with imaging to guide timely referrals, diagnostic imaging, and early interventions. Key therapeutic approaches include early referrals to rheumatology for optimizing systemic treatment, physiotherapy to maintain mandibular mobility, and orthodontic consultations in select cases. This comprehensive approach could be integrated into existing management protocols for JIA.

The relatively small sample size and the single-center study design may have limited the statistical power and generalizability of the results. While a statistically significant difference in mandibular mobility was observed between the groups, the ROC analysis did not show sufficient discriminant accuracy (AUC < 0.5). This indicates that there may be considerable overlap in clinical features between patients with and without temporomandibular arthritis, which could reduce diagnostic precision. This finding underscores the exploratory nature of the study, necessitating cautious interpretation of these preliminary results. Future multicenter studies with larger and more diverse cohorts are needed to confirm these observations, refine the diagnostic thresholds for mandibular mobility, and evaluate their clinical utility using standard pediatric assessment protocols. Additionally, prospective longitudinal studies should be conducted to ascertain whether functional markers, such as reduced mandibular mobility, can predict future imaging changes or craniofacial developmental disorders. Gathering such evidence would provide a more robust foundation for early diagnostic and therapeutic interventions in JIA children.

## 7. Conclusions

This cross-sectional clinical assessment suggests that reduced mobility in the jaw may be an indicator of TMJ arthritis in children with JIA. However, the findings should be approached with caution due to some methodological limitations. To confirm these results, larger multicenter studies utilizing validated pediatric assessment tools and standardized imaging techniques are necessary.

## Figures and Tables

**Table 1:** Characteristic of study participants.

Parameters		All JIA Patients	TMJ Arthritis Patients	Non-TMJ Arthritis Patients
		% of Patients/Age (years)		
**Gender**	female	67.50	69.67	64.70
	male	32.50	30.33	35.30
**Age**		12.87 ± 3.39	12.17 ± 3.65	13.82 ± 2.83
**Age at the diagnosis**		8.92 ± 3.68	8.13 ± 3.67	10.00 ± 3.51
**Duration of the disease**		3.95 ± 2.40	4.04 ± 2.72	3.82 ± 1.94
**Type of JIA**	oligoarthritis	47.5	52.17	41.18
	polyarthritis	52.5	47.83	58.82

## Data Availability

Data are available on request from the corresponding author.
